# Video-Assisted vs Robotic-Assisted Lung Lobectomies for Operating Room Resource Utilization and Patient Outcomes

**DOI:** 10.1001/jamanetworkopen.2024.8881

**Published:** 2024-05-03

**Authors:** Haley I. Tupper, Brian L. Lawson, Patricia Kipnis, Ashish R. Patel, Simon K. Ashiku, Nareg H. Roubinian, Laura C. Myers, Vincent X. Liu, Jeffrey B. Velotta

**Affiliations:** 1 Division of General Surgery, Department of Surgery, University of California, Los Angeles; 2 Division of Research, Kaiser Permanente Northern California, Oakland; 3 Division of Thoracic Surgery, Department of Surgery, Kaiser Permanente Oakland, Oakland, California; 4 Kaiser Permanente Bernard J. Tyson School of Medicine, Pasadena, California; 5 Department of Surgery, University of California San Francisco School of Medicine

## Abstract

**Importance:**

With increased use of robots, there is an inadequate understanding of minimally invasive modalities’ time costs. This study evaluates the operative durations of robotic-assisted vs video-assisted lung lobectomies.

**Objective:**

To compare resource utilization, specifically operative time, between video-assisted and robotic-assisted thoracoscopic lung lobectomies.

**Design, Setting, and Participants:**

This retrospective cohort study evaluated patients aged 18 to 90 years who underwent minimally invasive (robotic-assisted or video-assisted) lung lobectomy from January 1, 2020, to December 31, 2022, with 90 days’ follow-up after surgery. The study included multicenter electronic health record data from 21 hospitals within an integrated health care system in Northern California. Thoracic surgery was regionalized to 4 centers with 14 board-certified general thoracic surgeons.

**Exposures:**

Robotic-assisted or video-assisted lung lobectomy.

**Main Outcomes and Measures:**

The primary outcome was operative duration (cut to close) in minutes. Secondary outcomes were length of stay, 30-day readmission, and 90-day mortality. Comparisons between video-assisted and robotic-assisted lobectomies were generated using the Wilcoxon rank sum test for continuous variables and the χ^2^ test for categorical variables. The average treatment effects were estimated with augmented inverse probability treatment weighting (AIPTW). Patient and surgeon covariates were adjusted for and included patient demographics, comorbidities, and case complexity (age, sex, race and ethnicity, neighborhood deprivation index, body mass index, Charlson Comorbidity Index score, nonelective hospitalizations, emergency department visits, a validated laboratory derangement score, a validated institutional comorbidity score, a surgeon-designated complexity indicator, and a procedural code count), and a primary surgeon-specific indicator.

**Results:**

The study included 1088 patients (median age, 70.1 years [IQR, 63.3-75.8 years]; 704 [64.7%] female), of whom 446 (41.0%) underwent robotic-assisted and 642 (59.0%) underwent video-assisted lobectomy. The median unadjusted operative duration was 172.0 minutes (IQR, 128.0-226.0 minutes). After AIPTW, there was less than a 10% difference in all covariates between groups, and operative duration was a median 20.6 minutes (95% CI, 12.9-28.2 minutes; *P* < .001) longer for robotic-assisted compared with video-assisted lobectomies. There was no difference in adjusted secondary patient outcomes, specifically for length of stay (0.3 days; 95% CI, −0.3 to 0.8 days; *P* = .11) or risk of 30-day readmission (adjusted odds ratio, 1.29; 95% CI, 0.84-1.98; *P* = .13). The unadjusted 90-day mortality rate (1.3% [n = 14]) was too low for the AIPTW modeling process.

**Conclusions and Relevance:**

In this cohort study, there was no difference in patient outcomes between modalities, but operative duration was longer in robotic-assisted compared with video-assisted lung lobectomy. Given that this elevated operative duration is additive when applied systematically, increased consideration of appropriate patient selection for robotic-assisted lung lobectomy is needed to improve resource utilization.

## Key Points

**Question:**

Does resource utilization, particularly operative duration (from cut to close), differ between video-assisted and robotic-assisted thoracoscopic lung lobectomies in a community-based health system?

**Findings:**

This cohort study of 1088 patients aged 18 to 90 years found that the median robotic-assisted adjusted operative duration was 20.6 minutes longer than that for video-assisted lobectomy.

**Meaning:**

The findings of this study suggest that resource utilization, specifically operative duration, may be higher in robotic-assisted vs video-assisted lung lobectomies and that video-assisted lobectomies may be a more efficient use of surgical resources.

## Teaser

This cohort study assesses whether operating room (OR) resource utilization, in particular operative duration, differs between video-assisted and robotic-assisted thoracoscopic lung lobectomy among adult patients.

## Introduction

Pulmonary lobectomy is the most common thoracic operation.^1^ Lobectomies are overwhelmingly performed for lung cancer, the leading cause of cancer mortality in the United States,^2^ with national estimates indicating that over 40 000 lung cancer lobectomies were performed in 2017.^1^ Over 80% of lobectomies are performed minimally invasively, either robotic assisted or video assisted.^3^ Minimally invasive techniques are particularly suited to high-volume, excisional procedures^4^ and offer multiple advantages over open techniques, including decreased blood loss, less respiratory compromise, shorter length of stay, and reduced postoperative pain and complications, with equivalent oncologic outcomes.^5^

The proportion of minimally invasive lobectomies performed robotically has steadily increased with robotic techniques becoming an integral part of present-day thoracic training.^6^ The robot was initially developed to perform remote surgeries, a practical application which has stalled,^4^ but the technology offers superhuman dexterity with microscaling of movements and enhanced 3-dimensional visualization, which may be particularly advantageous in complex reconstructive procedures.^4^ Despite these advantages, multiple studies found no difference in key patient outcomes between video-assisted and robotic-assisted lung lobectomy.^7,8,9,10,11^ As with most new technology, costs are routinely higher for robotic-assisted surgeries compared with other surgeries,^5,7,8,10,12,13,14^ and earlier studies hinted at extended operative times.^5,15,16^ However, at the time of these studies, there had been insufficient robotic uptake to reach a learning-curve plateau. Between 2013 and 2018, the number of robotic-assisted lobectomies increased 243%.^6^

Over half of all minimally invasive lung lobectomies are now robotic assisted.^3^ Now is the opportune time to compare the operative efficiency of minimally invasive modalities, specifically video-assisted vs robotic-assisted lobectomies. Health care resource utilization is becoming increasingly relevant, and general thoracic surgery faces a substantial projected supply and demand imbalance: caseload is anticipated to increase 70% by 2035 from population growth and aging^17^ with another 37% increase from improved lung cancer–screening penetration.^18^ Meanwhile, the number of thoracic surgeons may decrease by over one-third over the same time period with operating room nursing shortages further compounding supply-side issues.^17,19^ Beyond resource considerations, prolonged operations are deleterious for patient outcomes.^20,21,22,23,24^ Consequently, the purpose of this multicenter retrospective cohort study was to compare resource utilization, particularly operative time, between video-assisted and robotic-assisted thoracoscopic lung lobectomies.

## Methods

This data-only study was approved by the Kaiser Permanente Northern California (KPNC) Institutional Review Board for the Protection of Human Subjects, which waived the individual informed consent requirement because the research posed no more than a minimum risk and could not be practically carried out without the waiver. Kaiser Permanente Northern California cares for approximately 4.5 million members under a mutual exclusivity arrangement. All 21 KPNC hospitals use the same information systems with a common medical record number.^25,26,27,28^ In this multicenter health system, thoracic surgery care is regionalized, meaning that all patients needing thoracic surgery are referred to 4 hospital centers with dedicated thoracic surgeons. This study followed the Strengthening the Reporting of Observational Studies in Epidemiology (STROBE) reporting guideline.

### Study Design

This was a retrospective cohort study of adult patients (18-90 years) who underwent a minimally invasive lung lobectomy between January 1, 2020, and December 31, 2022, at KPNC, a multicenter integrated hospital system with 14 board-certified general thoracic surgeons (A.R.P., S.K.A., and J.B.V.). Minimally invasive lobectomies were defined as either video-assisted thoracoscopic surgeries or robotic-assisted thoracoscopic surgeries and were identified using institutional procedural codes. All attending surgeons identified as primary surgeons were reviewed, and procedures performed by pediatric surgeons as the primary surgeon were excluded from analysis. Covariate and outcome data were extracted from KPNC electronic health record (EHR) databases for all patients from 1 year prior to surgery or the date of KPNC insurance enrollment, whichever was later, through 90 days after surgery.

### Covariates

We included the following covariates, which we defined on the date of surgery: sociodemographic variables, clinical variables, case-complexity variables, and a surgeon-specific indicator. Sociodemographic variables included age, sex, race and ethnicity, and neighborhood deprivation index (NDI).^29^ Self-reported race and Hispanic ethnicity were separately documented by clerks upon admission, but were aggregately reported in our study; race was not reported if a patient identified as Hispanic. Race categories included Asian, Black, White, and other (including American Indian or Alaska Native, Native Hawaiian or Other Pacific Islander, multiracial, or unknown). Race and ethnicity were included as a crude proxy for social inequalities impacting health, not accounted for by the NDI. The NDI in this study compares census block-group socioeconomic deprivation using American Community Survey data from 2020. For the NDI, 0 is the median, and negative numbers represent less deprivation. Clinical variables included patient body mass index, Charlson Comorbidity Index score (range, 0 to 24, with higher scores indicating greater morbidity; the maximum for patients in the study was 15), number of nonelective hospitalizations in the past year, number of emergency department treat-and-release episodes in the past year, and an abbreviated laboratory acuity score (Laboratory-Based Acute Physiology Score [abLAPS]).^30,31^ The abLAPS is an outpatient modification of a previously reported hospital score based on the lowest (indicating maximal physiologic derangement) score for 14 laboratory test results over the 30 days prior to the index date. In this study, the abLAPS was categorized into 4 categories: low (0-4), medium (5-10), high (>10), and none, based on 30-day preoperative laboratory results. None represents scenarios in which there was no clinical indication for these laboratory results. Specific case-complexity variables included the Comorbidity Assessment for Surgical Triage (CAST) score, which is a risk-stratification score for adverse surgical outcomes,^32^ a surgeon-designated patient medical-complexity indicator, and the total number of procedures performed during the operation. The CAST score incorporates patient and surgical case characteristics to predict the risk of 30-day postoperative morbidity, defined as a composite of mortality and major complications defined by the National Surgical Quality Improvement Program. The following inpatient CAST threshold probabilities were used for categorization: low, less than 2%; medium, 2% to less than 11%; and high, 11% or more. The number of procedures performed during the operation was based on institutional procedural codes. Case-complexity designation was entered as part of the surgical case request form in the EHR. During case-request submission, the surgeon classified the case as no perioperative medicine appointment required, relatively healthy, or relatively complex. Due to low frequency, no perioperative medicine appointment required was collapsed into relatively healthy. In the general surgical population, surgeons classify approximately 30% of cases as relatively complex. Surgical cases with the indication *cancer* or *within 2 weeks* are generally oncologic resections, while those with the indication *elective* typically are not cancer cases. There were no missing data for covariates.

### Outcomes

The primary outcome was overall operative (cut-to-close) duration in minutes. Secondary outcomes included patient hospital bed wheels-in-room time in minutes (room time), hospital length of stay in days, nonelective hospitalization within 30 days of discharge (hospital admission starting in the emergency department), and 90-day mortality. Mortality and nonelective hospitalization included data captured in the KPNC system as well as external systems, including state and federal reporting systems for mortality. There were no missing data for operative duration or length of stay. Given a time lag for federal and state mortality reporting, post hoc calculations suggested that we were missing between zero and five 90-day deaths (0%-0.5%).

### Statistical Analysis

We generated descriptive statistics and unadjusted comparisons between video-assisted and robotic-assisted lobectomies using the Wilcoxon rank sum test for continuous variables and the χ^2^ test for categorical variables. The study was estimated to have 80% power to detect a difference of 11 minutes or more in operative time between video-assisted and robotic-assisted surgeries. We estimated the average treatment effect of video-assisted vs robotic-assisted surgery using the augmented inverse probability of treatment weighting (AIPTW).^33^ Both the propensity and outcome models combined 5 learners using stacking, including the generalized linear model, the generalized additive model (gam package, version 1.22-3; R Project for Statistical Computing),^34^ multivariate adaptive regression splines (R package earth, version 5.3.2; R Project for Statistical Computing),^35^ random forests (ranger package, version 0.14.1; R Project for Statistical Computing),^36^ and extreme gradient boosting (xgboost, version 1.7.3.1).^37^ The SEs for estimates of the median treatment effect were based on 3-fold cross-fitting. Both propensity and outcome models used the covariates described above, with attending surgeon indicators added to the outcomes model to control for surgeon-to-surgeon variation.

We also conducted a sensitivity analysis to account for skills mastery. For this analysis, we reviewed all robotic-assisted and video-assisted lobectomies that each surgeon performed at KPNC from 2007 to 2022; we removed the first 25 robotic-assisted lobectomies and the first 25 video-assisted lobectomies that a surgeon performed at KPNC if any of those surgeries took place during the study period (2020-2022). The earliest year available in the EHR was 2007. We also performed a subanalysis comparing adjusted outcomes for surgeons who performed only robotic-assisted lobectomies with those who performed only video-assisted lobectomies. Analyses were performed using R, version 4.0.2 (R Project for Statistical Computing) and SAS, version 9.4 (SAS Institute Inc). A 2-sided *P* < .05 was considered significant.

## Results

A total of 1088 patients were analyzed (median age, 70.1 years [IQR, 63.3-75.8 years]; 704 female [64.7%] and 384 male [35.3%]). Patient characteristics were balanced between the 2 groups on all measures except Hispanic ethnicity, with more Hispanic patients undergoing robotic-assisted than video-assisted lobectomy (60 [13.5%] vs 51 [7.9%]) (Table 1). Of the total patients, 218 (20.0%) were Asian, 75 (6.9%) Black, 11 (10.2%) Hispanic, 644 (59.2%) White, and 40 (3.7%) of other race; 446 (41.0%) underwent robotic-assisted, and 642 (59.0%) underwent video-assisted lobectomy.

**Table 1.** Patient Characteristics by Minimally Invasive Modality

Characteristic	Patients, No. (%)			***P* value**
	Overall (N = 1088)	Robotic-assisted surgery (n = 446)	Video-assisted surgery (n = 642)	
**Sociodemographic variables**				
Age, median (IQR), y	70.1 (63.3-75.8)	70.9 (63.9-76.1)	69.7 (62.9-75.4)	.13
Sex				
Female	704 (64.7)	292 (65.5)	412 (64.2)	.71
Male	384 (35.3)	154 (34.5)	230 (35.8)	
Race and ethnicity				
Asian	218 (20.0)	86 (19.3)	132 (20.6)	.04
Black	75 (6.9)	25 (5.6)	50 (7.8)	
Hispanic^a^	111 (10.2)	60 (13.5)	51 (7.9)	
White	644 (59.2)	260 (58.3)	384 (59.8)	
Other^b^	40 (3.7)	15 (3.4)	25 (3.9)	
Neighborhood deprivation index, median (IQR)^c^	−0.5 (−0.9 to 0.1)	−0.5 (−0.9 to 0.2)	−0.5 (−0.9 to 0.1)	.31
**Clinical variables**				
BMI, median (IQR)	26.8 (23.5-30.5)	26.6 (23.4-30.5)	26.9 (23.5-30.5)	.81
Charlson Comorbidity Index score, median (IQR)	4.0 (3.0-7.0)	5.0 (3.0-7.0)	4.0 (3.0-7.0)	.41
Ever experienced in 1 y prior to surgery				
Nonelective hospitalization	162 (14.9)	76 (17.0)	86 (13.4)	.12
ED treat and release	339 (31.2)	146 (32.7)	193 (30.1)	.38
abLAPS^d^				
High	78 (7.2)	30 (6.7)	48 (7.5)	.77
Medium	98 (9.0)	39 (8.7)	59 (9.2)	
Low	323 (29.7)	140 (31.4)	183 (28.5)	
None	589 (54.1)	237 (53.1)	352 (54.8)	
**Surgical-case complexity variables**				
Relatively complex surgical case^e^	720 (66.2)	305 (68.4)	415 (64.6)	.22
CAST score^f^				
High	237 (21.8)	105 (23.5)	132 (20.6)	.28
Medium	839 (77.1)	338 (75.8)	501 (78.0)	
Low	12 (1.1)	3 (0.7)	9 (1.4)	
Associated *CPT* codes, median (IQR), No.	3.0 (2.0-3.0)	3.0 (2.0-3.0)	3.0 (2.0-3.0)	.99
Indication^g^				
Cancer	1051 (96.6)	437 (98.0)	614 (95.6)	.05
Elective	37 (3.4)	9 (2.0)	28 (4.4)	

Abbreviations: abLAPS, abbreviated Laboratory-Based Acute Physiology Score; BMI, body mass index (calculated as weight in kilograms divided by height in meters squared); CAST, Comorbidity Assessment for Surgical Triage; *CPT*, *Current Procedural Terminology*; ED, emergency department.

^a^ Independent of race.

^b^ Includes American Indian or Alaska Native, Native Hawaiian or Other Pacific Islander, multiracial, or unknown race.

^c^ The average is 0; negative numbers indicate less deprivation.

^d^ Scores range from no clinical indication for laboratory results (none), 0 to 4 (low), 5 to 10 (medium), and greater than 10 (high), with lower scores indicating maximum physiologic derangement in 30 days prior to surgery.

^e^ Yes or no designation by surgeon.

^f^ Scores range from less than 2% (low), 2% to less than 11% (medium), and 11% or more (high), with higher scores indicating greater probability of 30-day postoperative morbidity.

^g^ Variable not included in propensity model.

Patients had a median body mass index of 26.8 (IQR, 23.5-30.5) (calculated as weight in kilograms divided by height in meters squared), high degrees of multimorbidity (median Charlson Comorbidity Index score, 4.0 [IQR, 3.0-7.0]), and an overall medium postprocedural morbidity risk (839 patients [77.1%] with medium-risk CAST). In the year preceding surgery, 162 patients (14.9%) had 1 or more nonelective hospitalizations, and 339 (31.2%) had at least 1 emergency department treat-and-release encounter. Most patients had relatively normal laboratory values in the 30 days before the procedure (abLAPS low: 29.7% [n = 323]) or none at all (abLAPS none: 54.1% [n = 589]). Surgeons coded a median of 3.0 (IQR, 2.0-3.0) procedural codes per surgery. The 5 most commonly coded adjunctive procedures, in addition to a robotic-assisted or a video-assisted lobectomy, were consistent across modalities and included bronchoscopy, fiberoptic bronchoscopy, mediastinal dissection, mediastinal node dissection, and lung-wedge resection. Most surgeries (96.6% [n = 1051]) were for cancer.

The 14 board-certified thoracic surgeons generally demonstrated a preference for either robotic-assisted or video-assisted lung lobectomy, and half exclusively used 1 minimally invasive modality during the study period; 2 exclusively performed robotic-assisted lobectomies, while 5 exclusively performed video-assisted lobectomies. Among the surgeons who used both modalities, 5 performed more robotic-assisted lobectomies. Two surgeons who exclusively performed video-assisted lobectomies had particularly high-volume practices, performing 335 (30.8%) of all surgeries.

### Patient Characteristics by Minimally Invasive Modality

The median operative duration was 172.0 minutes (IQR, 128.0-226.0 minutes) with a median of 55.0 additional minutes (IQR, 46.0-65.0 minutes) of nonoperative patient wheels-in-room time (median room time: 231.0 minutes [IQR, 183.0-284.0 minutes]) (Table 2). Operative duration and hospital length of stay were longer for robotic-assisted compared with video-assisted surgeries. The median unadjusted operative duration of video-assisted lobectomies was 147.0 minutes (IQR, 107.0-198.0 minutes) compared with 204.0 minutes (IQR, 165.0-257.0 minutes) for robotic-assisted lobectomies (*P* < .001). The median length of stay for video-assisted surgeries was 2.2 days (IQR, 1.3-2.6 days) and 2.3 days (IQR, 1.5-3.3 days) for robotic-assisted surgeries (*P* < .001). Overall, 94 patients (8.6%) were readmitted within 30 days, and this did not differ between the 2 groups. The unadjusted 90-day mortality rate (1.3% [n = 14]) was too low for the AIPTW modeling process.

**Table 2.** Unadjusted Key Outcomes by Minimally Invasive Modality

Outcome	Modality			***P* value**
	Overall (N = 1088)	Robotic-assisted surgery (n = 446)	Video-assisted surgery (n = 642)	
Key outcomes				
Resource utilization, median (IQR), min				
Operative duration (cut to close)	172.0 (128.0-226.0)	204.0 (165.0-257.0)	147.0 (107.0-198.0)	<.001
Wheels-in-room time	231.0 (183.0-284.0)	264.5 (225.0-315.5)	203.0 (164.3-253.0)	<.001
Patient outcomes				
Hospital LOS, median (IQR), d	2.2 (1.4-3.2)	2.3 (1.5-3.3)	2.2 (1.3-2.6)	<.001
30-d Readmission rate, No. (%)	94 (8.6)	46 (10.3)	48 (7.5)	.13
90-d Mortality, No. (%)	14 (1.3)	5 (1.1)	9 (1.4)	.90

Abbreviation: LOS, length of stay.

### Adjusted Key Outcomes by Minimally Invasive Modality

After adjusting the sample using augmented inverse probability weights, there was less than a 10% difference in all covariates between robotic-assisted and video-assisted thoracoscopic lobectomies. The adjusted operative duration difference between the 2 invasive modalities was a median 20.6 minutes (95% CI, 12.9-28.2 minutes; *P* < .001), or 11.5% (Table 3). The robotic-assisted adjusted operative duration was 199.0 minutes (95% CI, 192.2-205.8 minutes) compared with 178.4 minutes (95% CI, 173.3-183.6 minutes) for video-assisted adjusted operative duration. The adjusted hospital bed wheels-in-room time was also longer for robotic-assisted lobectomies by 24.1 minutes (95% CI, 16.2-32.1 minutes). After adjustment, secondary outcome differences between the 2 groups, specifically hospital length of stay (0.3 days; 95% CI, −0.3 to 0.8 days; *P* = .11) and risk of 30-day readmission (adjusted odds ratio, 1.29; 95% CI, 0.84-1.98; *P* = .13), did not differ significantly.

**Table 3.** Adjusted Key Outcomes by Minimally Invasive Modality

Outcomes	Estimate (95% CI)		**Difference (95% CI) or adjusted odds ratio (95% CI)**	***P* value**
	Robotic-assisted surgery (n = 446)	Video-assisted surgery (n = 642)		
Resource utilization, min				
Operative duration (cut to close)	199.0 (192.2 to 205.8)	178.4 (173.3 to 183.6)	20.6 (12.9 to 28.2)	<.001
Wheels-in-room time	258.7 (251.8 to 265.7)	234.6 (229.3 to 239.9)	24.1 (16.2 to 32.1)	<.001
Patient outcomes				
Hospital LOS, d	3.0 (2.6 to 3.4)	2.7 (2.4 to 2.9)	0.3 (−0.3 to 0.8)	.11
Risk of 30-d readmission	0.10 (0.07 to 0.13)	0.08 (0.06 to 0.10)	1.29 (0.84 to 1.98)	.13

Abbreviation: LOS, length of stay.

On sensitivity analysis, excluding a surgeon’s first 25 surgeries, similar outcomes for operative duration were found (adjusted difference, 20.0 minutes; 95% CI, 11.1-28.8 minutes; *P* < .001) (eTable 1 in Supplement 1). The median length of stay was 0.60 days longer for robotic-assisted surgeries ([IQR, 0.01-1.20 days; *P* = .02), and 30-day nonelective readmission did not differ significantly between groups on adjusted analysis. When comparing adjusted outcomes for surgeons who performed only robotic-assisted lobectomies with those who performed only video-assisted lobectomies, the operative time difference persisted (adjusted difference, 25.1 minutes; 95% CI, 10.6-39.7 minutes; *P* < .001) (eTable 2 in Supplement 1).

## Discussion

Health care resources are finite and must be judiciously allocated. Even though surgical care is episodic, resource considerations in surgery are paramount given that surgical care accounts for almost one-third of US health care expenditure.^38^ The operating room is a particularly resource-intensive environment with operating room time as a key driver of resource utilization.^39,40,41^ The operating room time must be regarded as a precious shared resource.^39^ This cohort study of adult patients who underwent minimally invasive (robotic-assisted or video-assisted) lung lobectomy found that, in a community-based health care system with regionalization of thoracic surgery to 4 centers, robotic-assisted thoracoscopic lobectomies lasted a median 20.6 minutes, or 11.5%, longer than video-assisted thoracoscopic lobectomies without factoring in room setup or turnover time. Patient outcomes between approaches did not differ significantly.

Prior to conclusive evaluation of new surgical technologies and techniques, sufficient time must pass to allow for field penetration and skills mastery. Nearly 20 years were required for video-assisted lobectomy to become standard of care^42,43^ and to assuage concerns over cost and operative efficiency, despite decreased morbidity and equivalent oncologic outcomes.^15,44,45,46,47,48,49,50,51^ The first robotic lobectomy was in 2002.^52^ Sufficient time has passed for field penetration and skills mastery; it is finally appropriate to directly compare robotic-assisted and video-assisted lobectomies on patient outcome and resource utilization measures. For video-assisted thoracoscopic surgeries, increased operating room costs were negated by cost savings from shorter inpatient stays and associated with improved patient outcomes compared with open lobectomy.^13,48,49,50,51,53,54^ It is not clear that robotic-assisted lung lobectomy is broadly offering similar advantages over video-assisted lung lobectomy.^55^

Many studies compared robotic-assisted with open surgeries, but fewer directly compared minimally invasive modalities.^56^ The most replicable advantage of robotic-assisted compared with video-assisted thoracoscopic resection is increased hilar lymph node harvest.^9,10^ However, the amalgamation of studies has generally highlighted limited differences in patient outcomes between video-assisted and robotic-assisted surgeries.^7,8,9,10,16,57^ Earlier studies alluded to extended operative durations^24^ and increased resource utilization for robotic-assisted lobectomies, with 1 meta-analysis finding a 50-minute difference in operative duration,^11^ but these studies were conducted when robotic lobectomies were nascent.^58,59^ A recent industry-sponsored study highlighted that robotic-assisted lobectomies can be more expeditious in the hands of leading expert surgeons and institutions and may have fewer conversions.^60^ Results are still pending for an ongoing multicenter randomized clinical trial comparing robotic-assisted with video-assisted lobectomy in early-stage lung cancer, but preliminary microcosting results indicate that the incremental cost per quality-adjusted life-year is $14 926 higher for the robotic-assisted lobectomy.^61,62^ Our study supports prior results, finding significantly longer robotic-assisted vs video-assisted operative times in an era when robotic lobectomies are mainstream. These results were drawn from comprehensive, nationally representative multicenter data with a large number of thoracic surgeons and a high volume of lung lobectomies.^63^ Anecdotally, the decision to perform a lobectomy robotically in our system hinges on surgeon preference, which is informed by facility and ergonomics and robotic operating room availability. Unlike national databases, our study had the unique advantage of containing surgeon-specific information that allowed us to control for surgeon-to-surgeon variation.

Substantial energy has been devoted to optimizing operating room efficiency.^64^ Operating room time is expensive, costing an estimated $40 per minute inclusive of anesthesia.^39^ By these metrics, the time cost of robotic-assisted vs video-assisted lobectomy in the present study would have been an estimated $824 higher ($40 × 20.6 minutes). The majority of operating room costs are incurred from operating room staff salaries and overtime compensation.^39,65^ The number of hours that a surgeon operates in 1 day is independently associated with increased complications and length of stay for lung lobectomies.^66^ If a thoracic surgeon were to perform 3 robotic-assisted lobectomies in 1 day, their operative time may be 34.5% longer than if they were to perform video-assisted lobectomies, according to our study. Depending on staffing structures, the workdays of other health care professionals supporting the operating room may also be longer. Beyond financial repercussions, the most important impact of prolonged operative duration is the additional demand that it places on already-strained health systems. Prolonged or delayed surgical cases have a domino effect on other cases,^65^ and lack of operating room time is the leading cause of cancelled or delayed scheduled surgeries.^67^ For lung cancer, delaying surgical resection increases cancer recurrence risk and overall mortality.^68,69^ Operative efficiency is a key component of appropriate resource utilization and is an important consideration in alleviating health system strain.

### Limitations

Our study has limitations. The population was identified using procedural codes, which inherently relies on coding quality, although fastidious coding is incentivized in integrated payer-provider (payer-practitioner) systems. Non-KPNC 30-day readmissions could have been understated, but this is largely negated by mutual exclusivity care agreements and comprehensive capture of non-KPNC care for financial reasons. Our mortality estimates may have also been understated due to lags in the availability of non-KPNC data for mortality, but post hoc calculations suggest that we were only missing 0% to 0.5% of deaths. Despite the large size of this study, there were insufficient 90-day deaths to reliably report on this outcome with an adjusted analysis. Additional data must be gathered longitudinally or at institutional sites outside of Northern California to increase the sample size to adequately model 90-day mortality. Furthermore, although the clinical indication was relatively balanced between groups, we do not know if the clinical cancer stage differed between the 2 groups. We suspect no difference, as the choice of minimally invasive modality was largely driven by surgeon preference, but we do not have data to support this. Similarly, other relatively rare clinical factors that could prolong operative times, such as prior cardiothoracic surgery, were not included. The model accounted for many important patient and surgeon covariates, using a doubly robust method of adjustment (AIPTW), but we may have been missing relevant covariates. However, multiple well-validated, highly discriminatory composite variables were used in the study to accurately capture case complexity using granular data. Notably, this study was conducted when COVID-19 was maximally impacting the health system, which may have impacted operative durations for both modalities. Finally, the lack of bias in thoracic surgeon selection supported our study’s validity, but generalizability is a ubiquitous concern in studies involving surgical experience and technique. Further analysis is warranted to understand why robotic-assisted surgeries may be taking longer cut to close than video-assisted surgeries to assess for modifiable factors, including docking time. Future studies should also include operating room setup and turnover time to fully capture the time costs of different minimally invasive modalities. We plan to evaluate long-term survival in both groups.

## Conclusions

This cohort study of 1088 patients found no difference in patient outcomes between minimally invasive modalities, but operative duration was longer in robotic-assisted vs video-assisted lung lobectomy by 20.6 minutes after adjusting for multiple factors. The operating room is an expensive, resource-intensive environment, and efficient use must be a priority.^70^ Inefficient use can lead to case backlogs and delay other patients’ care. From a resource utilization perspective, this study preliminarily suggests that a robot should be used selectively. Further analyses are needed to understand when robotic-assisted lung lobectomies may offer a comparative advantage over video-assisted lung lobectomies to guide use in this relatively high-volume thoracic surgery.

## Notes

Supplement 1. **eTable 1.** Adjusted Sensitivity Analysis—Excludes Surgeon’s First 25 Institutional Surgeries **eTable 2.** Adjusted Sensitivity Analysis—Robotic-Only vs Video-Only Surgeons

Supplement 2.
Data Sharing Statement
