# Supplementary material for: Video-Assisted vs Robotic-Assisted Lung Lobectomies for Operating Room Resource Utilization and Patient Outcomes
Source: JAMA Netw Open. 2024 May 3;7(5):e248881. doi: 10.1001/jamanetworkopen.2024.8881 (PMC11069083; doi:10.1001/jamanetworkopen.2024.8881)
Supplement: Supplement 1. — eTable 1. Adjusted Sensitivity Analysis—Excludes Surgeon’s First 25 Institutional Surgeries eTable 2. Adjusted Sensitivity Analysis—Robotic-Only vs Video-Only Surgeons [file jamanetwopen-e248881-s001.pdf]

Supplemental Online Content

Tupper HI, Lawson BL, Kipnis P, et al. Video-assisted vs robotic-assisted lung lobectomies for operating room resource utilization and patient outcomes. *JAMA Netw Open.* 2024;7(5):e248881. doi:10.1001/jamanetworkopen.2024.8881

eTable 1. Adjusted Sensitivity Analysis—Excludes Surgeon's First 25 Institutional Surgeries

eTable 2. Adjusted Sensitivity Analysis—Robotic-Only vs Video-Only Surgeons

This supplemental material has been provided by the authors to give readers additional information about their work.

© 2024 Tupper HI et al. *JAMA Network Open.*

eTable 1. Adjusted Sensitivity Analysis—Excludes Surgeon's First 25 Institutional Surgeries

	**Robot**	**Video**		
Total	n = 274	n =593		
Key Outcomes	Estimate (95% CI)	Estimate (95% CI)	Difference or aOR (95% CI)	p
**Resource Utilization**				
Operative Duration (Cut-to-Close) in Minutes	195.2 (186.9-203.5)	175.2 (169.7-180.8)	20.0 (11.1-28.8)	<0.001
Wheels in Room Time in Minutes	252.5 (244.0-261.0)	230.7 (225.1-236.0)	21.8 (12.6-31.0)	<0.001
**Patient Outcomes**				
Hospital LOS in Days	3.2 (2.6-3.8)	2.6 (2.4-2.8)	0.6 (0.01-1.2)	0.02
Risk of 30-Day Readmission	0.08 (0.04-0.11)	0.08 (0.06-0.10)	1.02 (0.58-1.79)	0.47

eTable 2. Adjusted Sensitivity Analysis—Robotic-Only vs Video-Only Surgeons

	**Robot**	**Video**		
Total	n = 113	n = 452		
Key Outcomes	Estimate (95% CI)	Estimate (95% CI)	Difference or aOR (95% CI)	p
**Resource Utilization**				
Operative Duration (Cut-to-Close) in Minutes	181.0 (167.0-195.0)	155.8 (149.9-161.8	25.1 (10.6-39.7)	<0.001
Wheels in Room Time in Minutes	237.8 (221.8-253.8)	209.0 (203.0-215.0)	28.9 (12.3-45.4)	<0.001
**Patient Outcomes**				
Hospital LOS in Days	2.4 (2.0-2.7)	2.5 (2.3-2.7)	-0.1 (-0.5 to 0.3)	0.6
Risk of 30-Day Readmission	0.07 (0.02-0.11)	0.07 (0.04-0.09)	1.0 (0.5-2.3)	0.67

© 2024 Tupper HI et al. *JAMA Network Open.*
