# Supplementary material for: Video-Assisted vs Robotic-Assisted Lung Lobectomies for Operating Room Resource Utilization and Patient Outcomes
Source: JAMA Netw Open. 2024 May 3;7(5):e248881. doi: 10.1001/jamanetworkopen.2024.8881 (PMC11069083; doi:10.1001/jamanetworkopen.2024.8881)
Supplement: Supplement 2. — Data Sharing Statement [file jamanetwopen-e248881-s002.pdf]

Data Sharing Statement

Tupper. Video-Assisted vs Robotic-Assisted Lung Lobectomies for Operating Room Resource Utilization and Patient Outcomes. *JAMA Netw Open*. Published May 03, 2024. doi:10.1001/jamanetworkopen.2024.8881

Data
Data available: No

Additional Information
Explanation for why data not available: Per Kaiser Permanente Northern California policy, we are unable to share the data.
